# Dataset on investigating the effect of sunflower based biodiesel on the rheology of Nigeria waxy crude oil

**DOI:** 10.1016/j.dib.2018.08.106

**Published:** 2018-08-30

**Authors:** Adesina Fadairo, Temitope Ogunkunle, Victor Asuquo, Adebowale Oladepo, Babajide Lawal

**Affiliations:** a Covenant University, Ota, Nigeria; b PricewaterhouseCoopers, USA

## Abstract

This paper presents information about the data obtained from the experimental research showing the effect of sunflower based biodiesel on rheological properties of Nigeria waxy crude oil. The information reported in the dataset depicts 0.1–0.7% dosing concentration of sunflower based biodiesel might be required for viscosity reduction of Nigeria waxy crude oil at operational temperatures ranging from 10 °C (at low temperature region) to 60 °C (at mid temperature region). It has also demonstrated that biodiesel derived from sunflower is technically viable to decrease the viscosity of Nigeria waxy crude oil appreciably, hence revealing its potential capacity to enhancing flow of the oil in pipeline and wellbore system.

**Specifications Table**

**Table:** 

Subject area	*Petroleum Engineering*
More specific subject area	*Petroleum Production Engineering, Flow Assurance*
Type of data	*Table*
How data was acquired	OFITE viscometer.
Data format	*Raw and analyzed*
Experimental factors	Nigeria waxy crude was collected Niger- Delta region, pre-treated by heating the crude above 100 °C to remove water and possibly some contaminants and the properties of the crude were investigated. The sunflower oil was purchased, characterized and converted into biodiesel
Experimental features	The sunflower oil was characterized and converted into biodiesel by trans-esterification, the Nigeria waxy crude properties were also investigated upon the addition of sunflower based biodiesel at operational temperature of 10 °C to 60 °C.
Data source location	*Niger-Delta, Nigeria*
Data accessibility	Data are available within this article and student project of Department Petroleum Engineering, Covenant University, Nigeria
Related research article	*None*

**Value of the data**

• Data validate that the biodiesel gave a key performance indicator on the rheological properties of the Nigerian waxy crude especially at operational temperatures range from 10 °C to 60 °C.
• Data shows that viscosity of waxy crude oil decreases with addition of small concentrations of sunflower based biodiesel as additive at the same temperature.
• Data presented in this study is a pointer to show that sunflower based biodiesel is technically, environmentally and economically viable to serves as additive for enhancing flow of waxy crude in pipe.
• Data presented also shows that sunflower based biodiesel is one of the biodegradable agricultural product which can be applied as viscosity reducing and flow improver agent in oil and gas industry.

## 1 Data

Wax deposition is one of the most important challenges in the production of crude oil and production of hydrocarbon as reported in Ref. [1]. The problem generated by wax deposits is special concern in the production of crude oil at deep water where production fluid be cooled to nearly the temperature of the surrounding waters, hence decreasing the viscosity and retarding flow in pipe [2], [3], [4]. There is high demand of industry to achieve flow assurance throughout the production life of waxy crude oil. Experimental work in this data has shown that sunflower has technical potential to decrease the viscosity of Nigeria waxy crude oil appreciably.

Sunflower oil is mainly triglycerides (fats), typically derived from the fatty acids linoleic acid (which is doubly unsaturated) and oleic acid, a typical constituent is shown.

• Palmitic acid (saturated): 5%
• Stearic acid (saturated): 6%
• Oleic acid (monounsaturated omega-9): 30%
• Linoleic acid (polyunsaturated omega-6): 59%

Several other types of sunflower oils are produced, such as high linoleic, high oleic and mid oleic. Mid-oleic sunflower oil typically has at least 69% oleic acid. High oleic sunflower oil has at least 82% oleic acid. Variation in unsaturated fatty acids profile is strongly influenced by both genetics and climate. Table 1 shows the percentage of fatty acid composition by weight used as the basis for production of edible oil.

**Table 1:** Data on fatty acid composition of sunflower oil.

% Fatty acid composition By weight	16.01	18.00	20.00	22.00	24.00	18.01	22.01	18.02	18.03
Sunflower oil	6.08	3.26	0	0	0	16.9	0	73.7	0

### 1.1 Physical properties of the sunflower oil and the synthesis biodiesel

A documentation of the physical properties of the sunflower oil and the biodiesel derivative is presented in Table 2. The pour point, cloud point colour, saponification value are listed in the table The point at which the sunflower ceases to flow is given at −8.7 °C, while for the biodiesel is −18.9, this shows that biodiesel produced from sunflower oil is capable of delivering flow of waxy crude at very low temperatures and this is very good for the economics involved in production, transportation and storage of waxy crude in the facilities.

**Table 2:** Physical properties of sunflower oil and its biodiesel derivative.

Properties	Sunflower oil	Biodiesel derivative
Pour Point °C	−8.7	−18.9
Cloud Point °C	−6.5	−14.5
Flash Point °C	315	237
Saponification (mgKOH/g)	188–194	168–174
Acid value (mgKOH/g)	8.9	1.2
Viscosity (mm^2^/s)	189	2.39
Colour	Pale yellow	Golden yellow
Density (kg/m^3^) at 25 °C	918.8	838
Specific gravity	0.91	0.83

### 1.2 Properties and paraffin content of pure crude oil samples

Table 3 shows the various physical properties of the Nigerian waxy crude oil sample. The values determined were from the various experiments conducted in covenant university laboratory. The crude has a wax content of < 35% which means wax can precipitate at moderately low temperatures.

**Table 3:** Physical properties of crude oil sample.

Properties	Crude Sample
Specific gravity	0.847
API gravity(°API)	35.88
Pour Point(°C)	17.0
Plastic Viscosity(@40 °C)	12.0
Wax content (wt %)	27.2
Asphaltene content	0.17

### 1.3 Shear rate and shear stress at different concentrations

The crude oil sample exhibited the non-Newtonian fluid behaviour at lower temperatures for the pure crude sample, because the relationship between shear stress and shear rate is not constant. The shear stress of the crude sample was taken at different concentrations and at different temperatures.

Table 4, Table 5, Table 6, Table 7, Table 8 gives the data for the shear stress of the pure crude sample and the crude sample plus additives at different concentrations and at different temperatures, it is seen that the shear stress is decreasing as shear rates decreases and the shear stress decreases as temperature increases. The data shows the relationship of shear stress and shear rate for the pure crude sample and with the addition of biodiesel. The shear stress was higher in the pure crude sample, but with the addition of the biodiesel additives, the shear stress reduced giving an inverse relationship between the increase in concentration of the biodiesel additives and the shear stress.

**Table 4:** Shear stress for pure crude sample.

Shear rate (/s)	Shear stress (Pa s); Conc 0%		
	10 °C	20 °C	30 °C
1021.38	27.0671	20.428	10.214
510.69	18.8959	15.321	5.87305
340.46	17.8745	12.7675	4.85165
170.23	17.8745	9.7033	3.5749
102.138	15.06565	7.1498	2.80885

**Table 5:** Shear stress at 0.1 (v/v) concentration.

Shear rate (/s)	Shear stress (Pa s); Conc 0.1%				
	10 °C	20 °C	30 °C	40 °C	60 °C
1021.38	27.0671	19.9173	9.1926	6.6391	3.0642
510.69	16.3424	10.214	4.5963	4.0856	2.29815
340.46	16.3424	8.6819	3.83025	2.29815	2.0428
170.23	15.321	6.1284	1.78745	2.0428	1.78745
102.138	14.8103	4.5963	1.5321	1.27675	1.78745

**Table 6:** Shear stress at 0.3 (v/v) concentration.

Shear rate (/s)	Shear stress (Pa s); Conc 0.3%				
	10 °C	20 °C	30 °C	40 °C	60 °C
1021.38	20.428	15.321	8.1712	6.6391	3.5749
510.69	14.8103	11.2354	7.6605	4.5963	2.5535
340.46	12.7675	8.6819	4.5963	3.0642	2.29815
170.23	10.214	6.89445	3.83025	2.80885	2.29815
102.138	8.1712	4.34095	3.31955	2.0428	1.27675

**Table 7:** Shear stress at 0.5 (v/v) concentration.

Shear rate (/s)	Shear stress (Pa s); Conc 0.5%				
	10 °C	20 °C	30 °C	40 °C	60 °C
1021.38	13.7889	10.214	7.6605	6.1284	4.0856
510.69	12.2568	8.1712	4.34095	4.5963	2.5535
340.46	9.1926	6.1284	3.0642	3.83025	2.29815
170.23	8.6819	4.5963	1.78745	3.31955	2.0428
102.138	7.1498	3.0642	1.5321	2.0428	1.27675

**Table 8:** Shear stress at 0.7 (v/v) concentration.

Shear rate (/sec)	Shear stress (Pa s); Conc 0.7%				
	10 °C	20 °C	30 °C	40 °C	60 °C
1021.38	13.53355	10.214	6.1284	5.107	3.0642
510.69	11.2354	7.91585	4.0856	3.5749	2.0428
340.46	8.6819	6.1284	3.31955	3.31955	1.78745
170.23	8.6819	3.0642	2.0428	2.80885	1.78745
102.138	6.1284	3.0642	2.0428	1.78745	1.27675

#### 1.3.1 Shear rate of pure crude sample

See Table 4

#### 1.3.2 Shear stress of crude sample plus 0.1 (V/V) of biodiesel

See Table 5

#### 1.3.3 Shear stress of crude sample plus 0.3 (V/V) of biodiesel

 See Table 6

#### 1.3.4 Shear stress of crude sample plus 0.5 (V/V) of biodiesel

 See Table 7

#### 1.3.5 Shear stress of crude sample plus 0.7 (V/V) of biodiesel

 See Table 8

## 2 Experimental design, materials, and methods

### 2.1 Measurement of viscosity

Viscosity is the state of being thick, sticky, and semifluid in consistency, due to internal friction. It can also be defined as a measure of resistance of a fluid to flow. The measurement was carried out to investigate how flow behaves using the Nigerian waxy crude oil samples with additives and in base conditions at various temperatures. This was carried out using the OFITE viscometer

Procedure:

i. The Nigerian waxy crude oil was heated to a temperature of 45 °C and poured into the stainless steel cup, the sample cup was adjusted accordingly to fit in the holes on the viscometer.
ii. The sample cup was then set using the knot at the side of the viscometer such that the rotor sleeve was immersed in the sunflower oil up to the line indicated on the rotor sleeve enclosing the bob.
iii. The viscometer was switched on, the sample was first stirred for 10 s using the knob located at the top of the viscometer and the speed of the viscometer was set to 600 rpm and left for 10 s, then readings were checked and recorded from the scale deflection inside the viscometer. Subsequently corresponding readings was recorded for each of the samples at 300, 200, 100, 60 and 30 RPMs.

The crude oils samples were heated at high temperatures of 30 °C, 40 °C and 60 °C and also cooled to temperature between 10 °C to 20 °C to determine the behaviour of the crude oil samples at lower temperatures. Conversion of readings taken from the viscometer were converted to shear stress in Pascal by multiplying the crude oil samples by 0.5107 and the speeds were converted to shear rates in per second by multiplying the speeds in RPM by 1.7023.

#### 2.1.1 Impact of sunflower additives on the rheology of crude oil samples

The crude oil samples were doped with sunflower additives at several concentrations and the effect of the additives on the rheology of the crude oil samples at different temperatures.

300 mL of each of the samples were to be prepared and mixed with concentration of the additives from 0.1 (v/v) to 0.7 (v/v). The volume of crude was calculated to make 300 mL of samples and put into a steel cup with the different amount of additive added using a syringe and stirred to ensure a homogenous mixing of the additive and the crude oil sample. The rheology of the samples were then tested to analyse the impact of the additives.
