# Supplementary material for: Dataset on investigating the effect of sunflower based biodiesel on the rheology of Nigeria waxy crude oil
Source: Data Brief. 2018 Aug 30;20:948–53. doi: 10.1016/j.dib.2018.08.106 (PMC6138981; doi:10.1016/j.dib.2018.08.106)
Supplement: Supplementary file 1 — Supplementary material. [file mmc1.docx]

I wish to inform you that there is no any conflict of interest among the authors
